# HIPK2 restricts SIRT1 activity upon severe DNA damage by a phosphorylation-controlled mechanism

**DOI:** 10.1038/cdd.2015.75

**Published:** 2015-06-26

**Authors:** E Conrad, T Polonio-Vallon, M Meister, S Matt, N Bitomsky, C Herbel, M Liebl, V Greiner, B Kriznik, S Schumacher, E Krieghoff-Henning, T G Hofmann

**Affiliations:** 1 German Cancer Research Center (dkfz), Cellular Senescence Group, DKFZ-ZMBH Alliance, Im Neuenheimer Feld 280, 69120 Heidelberg, Germany

## Abstract

Upon severe DNA damage a cellular signalling network initiates a cell death response through activating tumour suppressor p53 in association with promyelocytic leukaemia (PML) nuclear bodies. The deacetylase Sirtuin 1 (SIRT1) suppresses cell death after DNA damage by antagonizing p53 acetylation. To facilitate efficient p53 acetylation, SIRT1 function needs to be restricted. How SIRT1 activity is regulated under these conditions remains largely unclear. Here we provide evidence that SIRT1 activity is limited upon severe DNA damage through phosphorylation by the DNA damage-responsive kinase HIPK2. We found that DNA damage provokes interaction of SIRT1 and HIPK2, which phosphorylates SIRT1 at Serine 682 upon lethal damage. Furthermore, upon DNA damage SIRT1 and HIPK2 colocalize at PML nuclear bodies, and PML depletion abrogates DNA damage-induced SIRT1 Ser682 phosphorylation. We show that Ser682 phosphorylation inhibits SIRT1 activity and impacts on p53 acetylation, apoptotic p53 target gene expression and cell death. Mechanistically, we found that DNA damage-induced SIRT1 Ser682 phosphorylation provokes disruption of the complex between SIRT1 and its activator AROS. Our findings indicate that phosphorylation-dependent restriction of SIRT1 activity by HIPK2 shapes the p53 response.

The evolutionarily conserved NAD^+^-dependent deacetylase Sirtuin 1 (SIRT1) has been linked to physiology and pathophysiology of central biological processes including cellular stress response, metabolic control, health-span regulation, genome stability, cell death regulation and cancer.^1, 2, 3, 4^ In accordance with its broad biological functions, a plethora of SIRT1 substrates have been identified. Through site-specific deacetylation of histones, SIRT1 has been linked to chromatin conformation, gene expression and regulation of the epigenetic landscape.^5, 6^ In addition, SIRT1 deacetylates and functionally regulates non-histone proteins including the transcription factors c-Myc, Foxo3, NF-κB, E2F1 and p53. ^7, 8, 9, 10, 11, 12, 13^

The p53 tumour suppressor is frequently mutated in human cancer cells.^14^ If not affected directly by gene mutation, frequent deregulation of the p53 activating/inactivating pathway is observed such as overexpression of its negative regulatory E3 ubiquitin ligase Mdm2. Upon genotoxic stress, p53 is stabilized, binds to specific target gene promoters and regulates gene sets, which specify cell fate towards apoptosis, senescence or DNA repair.^15, 16^ Stabilization of p53 in response to DNA damage requires posttranslational modifications including site-specific phosphorylation and acetylation.^15^ Acetylation of p53 by the acetyltransferases CBP and p300 has been shown to be indispensable for its apoptotic function.^17, 18^ SIRT1 antagonizes p53 acetylation, thereby suppressing apoptosis and facilitating cell survival after DNA damage.^3, 7, 13^ These findings argue that SIRT1 activity needs to be restricted upon severe, lethal DNA damage to achieve efficient p53 acetylation.

SIRT1 activity is regulated by various mechanisms including posttranslational modifications, intramolecular interaction as well as interaction with an inhibitor, deleted in breast cancer 1 (DBC1), or an activator, active regulator of SIRT1 (AROS).^19, 20, 21, 22, 23, 24, 25^ Previous reports show that ATM and ATR checkpoint kinases regulate SIRT1 function through phosphorylation of DBC1 at Thr454, which increases SIRT1-DBC1 binding.^24, 25^ Furthermore, SIRT1 is regulated in response to oxidative stress through phosphorylation at Ser27, Ser47 and Thr530 by the JNK1 kinase, which stimulates SIRT1 activity and nuclear localization.^26^

Tumour suppressor Homeodomain interacting protein kinase 2 (HIPK2) is a DNA damage-responsive cell fate regulator, which is negatively regulated by oncogenic signalling.^27, 28, 29, 30, 31, 32^ In undamaged cells HIPK2 is rapidly degraded by the ubiquitin ligase Siah-1.^33, 34, 35^ After DNA damage, HIPK2 is activated and stabilized through a concerted mechanism including autophosphorylation, recruitment of the phosphorylation-guided cis/trans isomerase Pin1 and ATM-mediated Siah-1 phosphorylation.^33, 36, 37^ Upon severe DNA damage HIPK2 phosphorylates p53 at Serine 46 in association with promyelocytic leukaemia (PML) nuclear bodies (NBs), and thus potentiates apoptotic target gene expression by p53.^38, 39, 40^ In addition, p53 stimulates PML expression and nuclear trafficking, providing an additional regulatory link between both proteins.^41, 42^ PML and its associated NBs play an important role in cell fate regulation, DNA damage-induced senescence and cell death.^43, 44, 45, 46^ By providing a catalytic surface for enzymes regulating p53 posttranslational modifications, PML-NBs modulate the phosphorylation pattern of p53 after genotoxic stress.^39, 43, 44, 46, 47, 48^ As both activators and inhibitors of p53 function localize to PML-NBs (such as HIPK2 and SIRT1, respectively), this argues that crosstalk between these regulators might shape p53 posttranslational modification and function.

Here we investigated the regulation of SIRT1 in response to DNA damage. We show that SIRT1 activity is restricted in response to severe DNA damage through phosphorylation of SIRT1 by HIPK2 at PML-NBs.

## Results

### SIRT1 and HIPK2 interact in response to DNA damage

**Figure 1:**
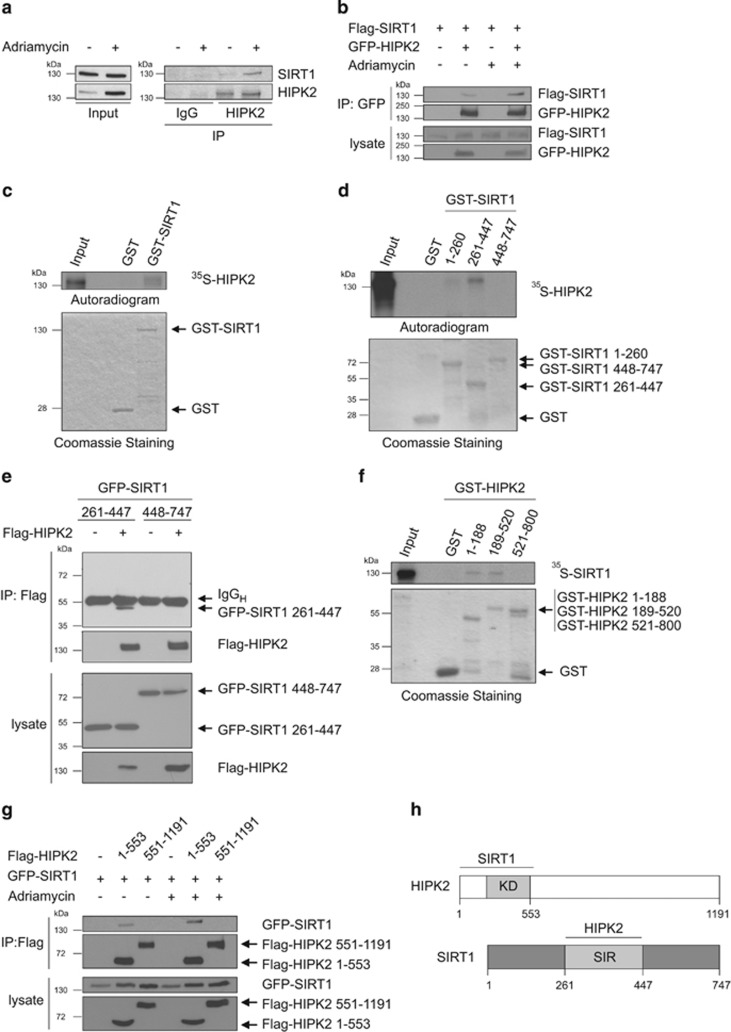
SIRT1 and HIPK2 interact in response to DNA damage. (**a**) Interaction of endogenous SIRT1 and HIPK2 upon DNA damage (0.75 *μ*g/ml Adriamycin for 24 h). HIPK2 was precipitated from U2OS cell lysates as indicated and coprecipitated SIRT1 was analysed by immunoblotting. The input control is 2% of the total cell lysate. (**b**) Interaction of ectopically expressed SIRT1 and HIPK2 after DNA damage. Flag-SIRT1 and GFP-HIPK2 were expressed in 293T cells. Twenty-four hours after transfection cells were treated with adriamycin (0.75 *μ*g/ml for 24 h) or left untreated. GFP-HIPK2 was precipitated from the lysates and the binding of Flag-SIRT1 to GFP-HIPK2 was measured by immunoblot analysis. The input control is 10% of total cell lysates. (**c**) *In vitro* interaction between SIRT1 and HIPK2. GST-SIRT1 and GST were incubated with *in vitro* translated ^35^S-HIPK2, GST pull-downs were performed and analysed by SDS–PAGE and autoradiography. Two percent input was loaded as input control. Total amounts of proteins were analysed by Coomassie Brilliant Blue staining. (**d**) GST pulldown assays were performed with recombinant GST-SIRT1 truncations and ^35^S-labelled HIPK2. Two percent input was loaded as control. (**e**) Flag-HIPK2 and the truncation mutants GFP-SIRT1 (aa 261–447) and (aa 448–747) were expressed in 293T cells. Flag-HIPK2 protein was precipitated from the lysates and co-immunoprecipitation of GFP-SIRT1 was analysed by immunoblot using the indicated antibodies. As input controls 5% of the total cell lysates were analysed. (**f**) GST-pulldown with recombinant GST-HIPK2 truncations and ^35^S-labelled SIRT1. In all, 2% input were loaded as input control. (**g**) GFP-SIRT1 and the truncation mutants Flag-HIPK2 (aa 1–553) and (aa 551–1191) were expressed in 293T cells. Twenty-four hours after transfection cells were incubated with Adriamycin (0.75 *μ*g/ml for 24 h) or left untreated. Flag-HIPK2 protein was precipitated from the lysates and co-immunoprecipitation of GFP-SIRT1 was analysed by immunoblot using the indicated antibodies. As input controls 10% of the total cell lysates were used. (**h**) Schematic representation of the SIRT1–HIPK2 interaction

SIRT1 and HIPK2 are known to localize to PML-NBs and to regulate p53 modification in response to genotoxic stress.^46^ To examine potential crosstalk between SIRT1 and HIPK2 we assessed complex formation of the endogenous proteins upon DNA damage generated by treatment with the chemotherapeutic drug Adriamycin. We used U2OS cells, a widely used cell model to study the DNA damage response and known to express endogenous HIPK2, SIRT1 and wild-type p53.^20, 33^ Immunoblot analysis revealed co-immunoprecipitation of endogenous SIRT1 and HIPK2 after Adriamycin treatment, indicating that SIRT1 and HIPK2 interact upon DNA damage (Figure 1a).

To further strengthen our findings, we studied complex formation of Flag-SIRT1 and GFP-HIPK2 ectopically expressed in 293T cells, a cell model allowing efficient transfection and protein expression. Coimmunoprecipitation analysis indicated weak interaction of overexpressed Flag-SIRT1 with GFP-HIPK2 (Figure 1b). Notably, association of Flag-SIRT1 and GFP-HIPK2 was clearly potentiated in response to Adriamycin treatment (Figure 1b), supporting the conclusion that SIRT1–HIPK2 complex formation is stimulated by DNA damage. Taken together, our results show that SIRT1 forms a complex with HIPK2 in response to DNA damage.

### Mapping of the interacting interfaces on SIRT1 and HIPK2

To characterise the interaction of SIRT1 and HIPK2 more detailed, we performed *in* *vitro* GST pulldown assays. To this end, we expressed a GST-SIRT1 fusion protein in *E.coli*, purified the protein and incubated it with *in vitro* ^35^S-labelled HIPK2. ^35^S-labelled HIPK2 was pulled-down with GST-SIRT1 but not with GST alone (Figure 1c), indicating that SIRT1 and HIPK2 interact *in vitro*, presumably in a direct fashion.

To map the HIPK2-interacting domain of SIRT1, we generated non-overlapping GST-SIRT1 deletions spanning the regulatory N-terminus (amino acids 1–260), the catalytic SIR domain (aa 261–447) and the regulatory C-terminus (aa 448–747) and performed GST pulldown assays. Our results revealed clear interaction of HIPK2 with the central, catalytic SIR domain (Figure 1d). No interaction of HIPK2 with the C-terminus and only faint interaction with the N-terminal regulatory domain of SIRT1 were detectable. These findings are further supported by co-immunoprecipitation analyses using ectopically expressed Flag-HIPK2 and GFP-SIRT1 truncation mutants (Figure 1e).

To define the SIRT1-interacting interface on HIPK2 we used GST-HIPK2 deletions and performed GST pulldown assays with ^35^S-labelled SIRT1. The results indicate that both the extreme N-terminus (aa 1–188) and the kinase domain (aa 189–520) of HIPK2 mediate interaction with SIRT1 (Figure 1f). To substantiate these findings we performed coimmunoprecipitation analyses using truncated Flag-HIPK2^1–553^ and Flag-HIPK2^551–1191^ along with full-length GFP-SIRT1. Weak binding between HIPK2^1–553^ and GFP-SIRT1 was observed in the absence of DNA damage, and the interaction was enhanced after DNA damage (Figure 1g). No interaction between GFP-SIRT1 and HIPK2^551–1191^ was observed (Figure 1g). In summary, our results indicate that SIRT1 binds via its central SIR domain to the N-terminus and the kinase domain of HIPK2 (Figure 1h).

### HIPK2 phosphorylates SIRT1 *in vitro*

**Figure 2:**
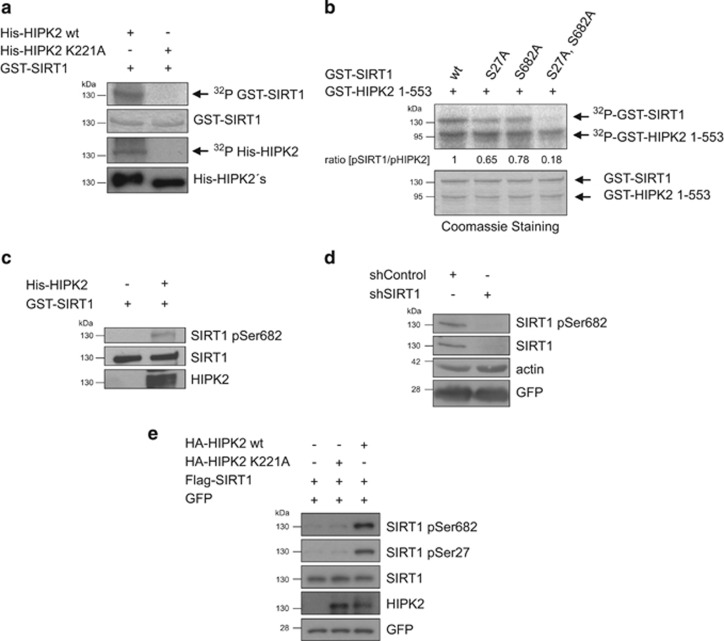
HIPK2 phosphorylates SIRT1 *in vitro* and in cellular context. (**a**) HIPK2 directly phosphorylates SIRT1. Recombinant 6xHis-HIPK2, kinase-deficient 6xHis-HIPK2^K221A^ and GST-SIRT1 were purified from *E. coli*. *In vitro* kinase assays were performed and SIRT1 phosphorylation as well as HIPK2 autophosphorylation was examined by SDS–PAGE and autoradiography. Protein levels were analysed by either Coomassie Brilliant Blue staining or immunoblotting. (**b**) HIPK2 phosphorylates SIRT1 at Ser27 and Ser682 *in vitro*. GST-SIRT1 wild type, SIRT1 S27A, SIRT1 S682A and the double mutant SIRT1 S27A, S682A were incubated with a truncated, catalytically active GST-HIPK2^1–553^. *In vitro* kinase assay was analysed by SDS–PAGE and autoradiography. Protein levels were analysed by Coomassie Brilliant Blue staining. The ratio (amount pSIRT1)/(amount pHIPK2) was quantified by densitometry using the ImageJ software. (**c**) Recombinant GST-SIRT1 and 6xHis-HIPK2 purified from *E.coli* were subjected to an *in vitro* kinase reaction. GST-SIRT1 phosphorylation was analysed by immunoblotting using the indicated antibodies. (**d**) U2OS cells were retrovirally transduced with a SIRT1-specific shRNA or a control shRNA. Depletion of SIRT1 was examined by immunoblot analysis using the indicated antibodies. The expression of GFP protein was used as a control for the transduction efficiency. (**e**) HIPK2 phosphorylates SIRT1 in cells. HA-HIPK2 wild type or kinase-deficient point mutant HA-HIPK2^K221A^ constructs were expressed along with Flag-SIRT1 in 293T cells. Twenty-four hours after transfection cell lysates were analysed by immunoblotting using the indicated antibodies

Since HIPK2 is a Ser/Thr-kinase, we next investigated whether SIRT1 is a HIPK2 substrate. To this end, we performed *in vitro* kinase assays by incubating bacterially expressed, purified GST-SIRT1 and His-HIPK2 proteins. Indeed, SIRT1 was phosphorylated by wild-type His-HIPK2 but not by a kinase-deficient His-HIPK2^K221A^ point mutant, demonstrating direct phosphorylation of SIRT1 by HIPK2 (Figure 2a).

To map the phosphorylation site(s) we used truncated GST-SIRT1 proteins and GST-SIRT1 proteins where we had exchanged Ser/Thr residues at potential HIPK2 target sites (Ser-Pro/Thr-Pro sites) to Ala. This approach identified two sites on SIRT1, Ser27 and Ser682, to be phosphorylated by HIPK2 (Supplementary Figure S1a and b).

Next, the phosphorylation sites were validated in the SIRT1 full-length context *in vitro*. Whereas single mutation of Ser27 or Ser682 moderately reduced SIRT1 phosphorylation, simultaneous mutation of both sites led to a profound reduction of SIRT1 phosphorylation by HIPK2 (Figure 2b), identifying these sites as major phosphorylation sites on SIRT1. Database analysis revealed a high degree of conservation of Ser682, in contrast to Ser27, which was found to be less conserved in evolution (Supplementary Figure S1c).

### Phosphorylation of SIRT1 at Ser682 by HIPK2

Next, we raised a phosphorylation-specific antibody against the Ser682 phospho-site of SIRT1 and affinity-purified it using the phospho-peptide. Immunoblot analysis showed that the antibody specifically recognized GST-SIRT1 phosphorylated by HIPK2, but not unphosphorylated SIRT1, validating its specificity towards Ser682-phosphorylated SIRT1 (Figure 2c). Furthermore, depletion of SIRT1 in U2OS cells by SIRT1-specific RNA interference abolished the signal of the SIRT1 pSer682 antibody (Figure 2d), indicating its specificity to SIRT1. Together, these results show that the antibody specifically recognizes Ser682-phosphorylated SIRT1.

We next studied Ser27 and Ser682 phosphorylation of ectopically expressed SIRT1. Immunoblot analysis using a commercially available phospho-specific SIRT1 pSer27 SIRT1 antibody and our SIRT1 pSer682 antibody revealed that phosphorylation of SIRT1 at Ser27 and Ser682 is stimulated by wild-type HIPK2 but not by its kinase-deficient form (Figure 2e), indicating that HIPK2 can phosphorylate both sites in the cellular context.

### HIPK2 phosphorylates SIRT1 at Ser682 after DNA damage

**Figure 3:**
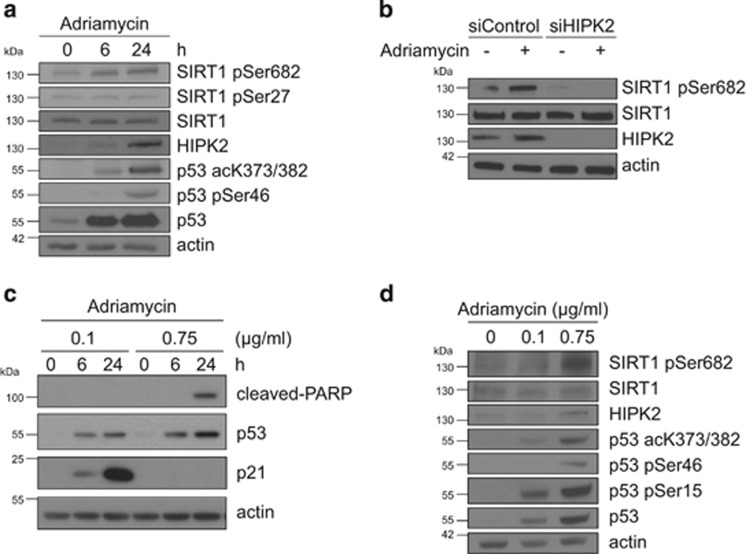
Phosphorylation of SIRT1 at Ser682 by HIPK2 upon DNA damage. (**a**) SIRT1 Ser682 phosphorylation is linked to DNA damage. U2OS cells were treated with 0.75 *μ*g/ml of Adriamycin for the indicated time points. Total cell lysates were analysed by immunoblotting using the indicated antibodies. (**b**) SIRT1 Ser682 phosphorylation depends on HIPK2. U2OS cells were transfected with HIPK2-targeted siRNA. Twenty-four hours post-transfection cells were treated with Adriamycin (0.75 *μ*g/ml) for 24 hours or left untreated. Total cell lysates were analysed by immunoblotting using the indicated antibodies. (**c**) U2OS cells were treated with sublethal (0.1 *μ*g/ml) and lethal (0.75 *μ*g/ml) doses of Adriamycin for the indicated time points. Total cell lysates were analysed by immunoblotting using the indicated antibodies. (**d**) SIRT1 Ser682 phosphorylation is linked to lethal DNA damage. U2OS cells were treated with sublethal (0.1 *μ*g/ml) and lethal (0.75 *μ*g/ml) doses of Adriamycin for 24 hours. Total cell lysates were analysed by immunoblotting using the indicated antibodies

To determine whether endogenous SIRT1 is phosphorylated at Ser27 and/or Ser682 in after DNA damage, we treated U2OS cells with 0.75 *μ*g/ml Adriamycin and analysed the lysates by immunoblotting. Although immunoblotting indicated weak background phosphorylation of SIRT1 at Ser682 in unstressed cells, a robust increase in SIRT1 Ser682 phosphorylation was observed upon Adriamycin treatment (Figure 3a). Furthermore, SIRT1 Ser682 phosphorylation correlated with HIPK2 stabilization, p53 Ser46 phosphorylation and acetylation of p53 at Lys382 (Figure 3a). Unexpectedly, we observed no increase in Ser27 phosphorylation of SIRT1 after DNA damage (Figure 3a). Thus, we focused our analyses on the regulation and function of SIRT1 Ser682 phosphorylation.

To investigate whether HIPK2 is essential for SIRT1 Ser682 phosphorylation upon DNA damage, we depleted endogenous HIPK2 by RNA interference. Whereas control siRNA-transfected cells showed HIPK2 stabilization and concomittant Ser682 phosphorylation of SIRT1 upon DNA damage (Figure 3b), HIPK2-depleted cells showed a strong reduction in SIRT1 Ser682 phosphorylation (Figure 3b), demonstrating that HIPK2 is essential for SIRT1 Ser682 phosphorylation after DNA damage.

### Phosphorylation of SIRT1 at Ser682 is linked to lethal DNA damage

We next studied whether SIRT1 is differentially phosphorylated at Ser682 after sublethal *vs* lethal DNA damage, similar to the p53 Ser46 phosphorylation mark, a HIPK2 target residue linked to lethal damage.^38, 39, 49^ To this end, we treated U2OS cells with 0.1 *μ*g/ml and 0.75 *μ*g/ml Adriamycin, respectively. FACS-based analysis in combination with immunoblotting for the cell cycle inhibitor p21 (which is downregulated after lethal damage^50^) and caspase-cleaved PARP confirmed 0.1 *μ*g/ml Adriamycin as sublethal and 0.75 *μ*g/ml Adriamycin as lethal dose (Figure 3c and Supplementary Figure S2). Interestingly, phosphorylation of SIRT1 at Ser682 was triggered in response to lethal DNA damage, but remained unresponsive to sublethal DNA damage (Figure 3d). In addition, phosphorylation of p53 at Ser46, an established marker for lethal DNA damage,^49^ correlated with SIRT1 Ser682 phosphorylation and was specifically induced after lethal damage (Figure 3d). Phosphorylation of p53 at Ser15 by ATM was detectable both after sublethal and lethal damage (Figure 3d), in accordance with previous studies.^37, 49^ Together, our findings demonstrate that phosphorylation of SIRT1 at Ser682 is linked to lethal DNA damage.

### DNA damage-induced phosphorylation of SIRT1 at Ser682 is linked to PML-NBs

**Figure 4:**
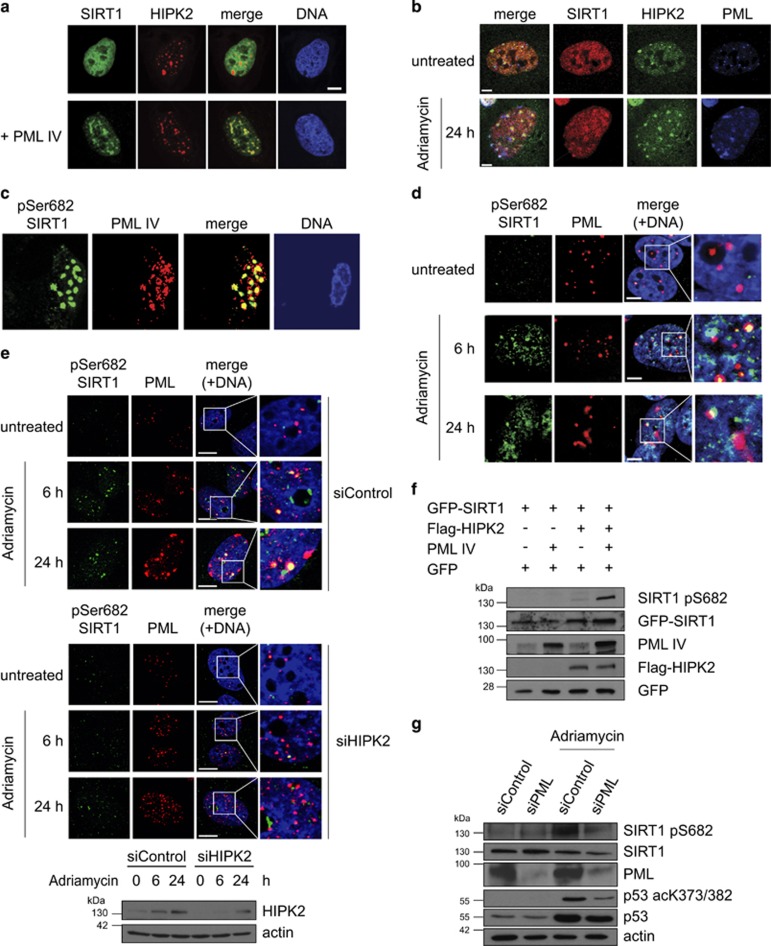
Regulation of SIRT1 Ser682 phosphorylation at PML-NBs. (**a**) PML provokes colocalization of SIRT1 and HIPK2 at PML-NBs. GFP-SIRT1 (green), mCherry-HIPK2 (red) (upper panels) and PML IV (lower panels) were co-expressed in U2OS cells and analysed by immunofluorescence staining and confocal microscopy. DNA is stained by Hoechst33342 (blue). Scale bar, 10 *μ*m. (**b**) Endogenous SIRT1 and HIPK2 co-localize at PML-NBs upon DNA damage. U2OS cells were treated with adriamyin (0.75 *μ*g/ml) for 24 h. Endogenous PML, HIPK2 and SIRT1 were stained using indirect immunofluorescence. Representative confocal images are shown. Scale bar, 5 *μ*m. (**c**) Phosphorylated SIRT1 localizes to PML-NBs. Flag-PML IV, SIRT1 and HA-HIPK2 were expressed in U2OS cells. pSer682 SIRT1 (anti-pSer682) and PML (anti-Flag) were examined by immunofluorescence staining and confocal microscopy. Scale bar, 10 *μ*m. DNA is visualized with Hoechst33342 (blue). Representative cells are shown. (**d**) Endogenous SIRT1 phosphorylated at Ser682 localizes upon DNA damage to PML-NBs. U2OS cells were treated with adriamyin (0.75 *μ*g/ml) for the indicated timepoints and analysed by indirect immunofluorescence staining. Representative confocal images are shown. Scale bar, 5 *μ*m. (**e**) Localization of phosphorylated SIRT1 to PML-NBs is dependent on HIPK2 expression. U2OS cells were transfected with HIPK2-targeted siRNA. Twenty-four hours post-transfection cells were treated with adriamycin (0.75 *μ*g/ml) for 6 and 24 h or left untreated. Total cell lysates were analysed by immunoblotting using the indicated antibodies. In parallel, cells were analysed by indirect immunofluorescence staining. Representative confocal images are shown. Scale bar, 5 *μ*m. (**f**) Ectopic expression of PML potentiates SIRT1 phosphorylation at Ser682. U2OS cells were transfected with the indicated constructs and cell lysates were analysed by immunoblotting using the indicated antibodies. (**g**) Depletion of endogenous PML expression by RNA interference results in diminished SIRT1 Ser682 phosphorylation after DNA damage. U2OS cell were treated with the indicated siRNA and treated with Adriamycin (0.75 *μ*g/ml) for 24 h. Total cell lysates were analysed by immunoblotting using the indicated antibodies

Both HIPK2 and SIRT1 can be recruited to PML-NBs upon stress.^39, 47^ In addition, PML-NBs have an important role in regulating p53 posttranslational modifications and pro-apoptotic function.^46, 51^ To address a potential interplay between SIRT1 and HIPK2 at PML-NBs we performed immunofluorescence stainings. Confocal microscopy revealed that ectopic expression of PML IV, a PML isoform known to recruit HIPK2 to PML-NBs,^39^ stimulated co-localization of SIRT1 and HIPK2 at PML-NBs (Figure 4a).

We next aimed to determine the subcellular localization of endogenous SIRT1, HIPK2 and PML proteins in the absence and presence of DNA damage. Confocal microscopy analyses indicated no substantial co-localization of SIRT1 and HIPK2 in unstressed cells (Figure 4b). However, after Adriamycin treatment, endogenous SIRT1 and HIPK2 co-localized at PML-NBs (Figure 4b), suggesting that HIPK2-mediated SIRT1 phosphorylation takes place in association with PML-NBs. Indeed, confocal microscopy indicated that Ser682-phosphorylated SIRT1 localizes to PML-NBs upon overexpression of PML IV (Figure 4c). Furthermore, also a fraction of endogenous SIRT1 phosphorylated at Ser682 localized to PML-NBs after DNA damage (Figure 4d), which is in line with the hypothesis that SIRT1 Ser682 phosphorylation takes place at PML-NBs.

To determine whether SIRT1 Ser682 phosphorylation at PML-NBs depends on HIPK2, we depleted endogenous HIPK2 by RNA interference and performed confocal microscopy. Robust reduction of the pSer682 SIRT1 signal at PML-NBs was observed upon HIPK2 depletion (Figure 4e), indicating that HIPK2 is essential for SIRT1 Ser682 phosphorylation at PML-NBs. Together, these findings support the conclusion that HIPK2 phosphorylates SIRT1 in association with PML-NBs.

We next analysed the role of PML in HIPK2-mediated SIRT1 phosphorylation. Interestingly, ectopic expression of PML IV substantially potentiated HIPK2-mediated SIRT1 Ser682 phosphorylation (Figure 4f). Accordingly, PML IV expression also enhanced HIPK2-SIRT1 interaction (Supplementary Figure S3).

We also depleted endogenous PML by RNA interference and analysed the effect on SIRT1 Ser682 phosphorylation. PML depletion resulted in a massive reduction of SIRT1 Ser682 phosphorylation after DNA damage (Figure 4g), demonstrating an essential role of PML in HIPK2-mediated SIRT1 phosphorylation. PML depletion also led to reduced p53 Lys382 acetylation (Figure 4g), which is in line with previous reports.^44, 48^ Collectively, our findings unveil an essential role of PML in HIPK2-mediated SIRT1 Ser682 phosphorylation.

### Ser682 phosphorylation inhibits SIRT1 activity and facilitates efficient p53 acetylation

**Figure 5:**
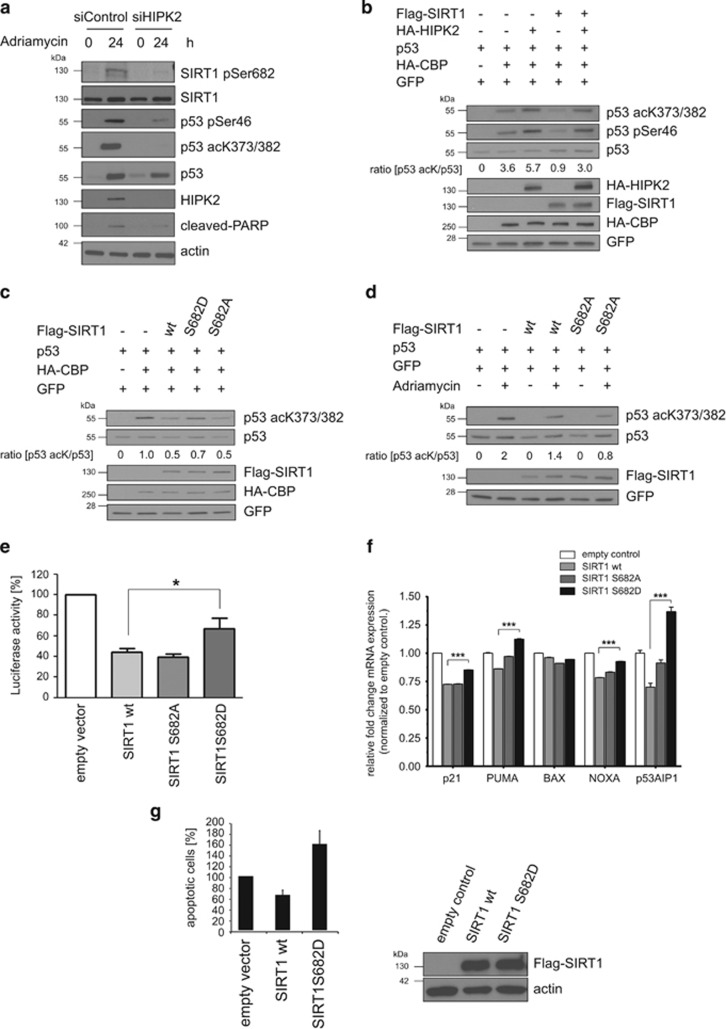
Ser682 phosphorylation regulates SIRT1 deacetylase activity, p53 acetylation and cell death. (**a**) HIPK2 is important for p53 Lys382 acetylation after DNA damage. HIPK2 was depleted in U2OS cells by RNA interference and cells were treated with Adriamycin (0.75 *μ*g/ml) as indicated. Total cell lysates were analysed by immunoblotting using the indicated antibodies. (**b**) HIPK2 antagonizes SIRT1-mediated deacetylation of p53. Flag-SIRT1, HA-HIPK2, p53 and HA-CBP were expressed in H1299 cells as indicated and total cell lysates were analysed by immunoblotting using the indicated antibodies. GFP expression was used to control the transfection efficiency. Total p53 protein expression levels were adjusted to equal levels to be able to compare p53 acetylation under different conditions. The ratio (amount p53 acK)/(amount p53) was quantified by densitometry using the ImageJ software. (**c**) SIRT1 Ser682 is required for HIPK2-mediated SIRT1 inhibition. H1299 cells were transfected with the indicated constructs and total cell lysates were analysed by immunoblotting using the indicated antibodies. GFP expression was used to control the transfection efficiency. Total p53 protein expression levels were adjusted to equal levels to be able to compare p53 acetylation under different conditions. The ratio (amount p53 acK)/(amount p53) was quantified by densitometry using the ImageJ software. (**d**) SIRT1 Ser682 is important for DNA damage-stimulated p53 acetylation. H1299 cells were transfected with the indicated expression constructs. Twenty-four hours after transfection cells were treated with Adriamycin (0.75 *μ*g/ml) or left untreated. Total cell lysates were analysed by immunoblotting. GFP expression was used to control the transfection efficiency. Total p53 protein expression levels were adjusted to equal levels to be able to compare p53 acetylation under different conditions. The ratio (amount p53 acK)/(amount p53) was quantified by densitometry using the ImageJ software. (**e**) SIRT1 Ser682 phosphorylation regulates p53-dependent transcription. U2OS cells were transfected with the indicated expression constructs together with a luciferase PUMA-reporter. Firefly reporter activity was normalized to Renilla reporter activity. Data are shown as means±S.D.; *n*=3; *P*<0.05, Student's *t*-test. (**f**) U2OS cells were transfected with the indicated expression constructs and expression of p53 target genes: p21, PUMA, BAX, NOXA and p53AIP1 were analysed by qRT-PCR. mRNA expression levels were normalized to the empty vector control. Data are shown as means±S.D.; *n*=3; *P*<0.05, Student's *t*-test. (**g**) SIRT1 Ser682 phorphorylation potentiates apoptosis. U2OS cells were transfected with the indicated expression constructs. Twenty-four hours after transfection cells were treated with Adriamycin (1 *μ*g/ml, 48 h) and subsequently analysed by FACS using Annexin V-FITC

To gain insight in the functional consequences of HIPK2-mediated SIRT1 phosphorylation, we analysed p53 acetylation, which is well-established to be negatively regulated by SIRT1.^7, 13^ First, we determined the relevance of endogenous HIPK2 expression in DNA damage-induced p53 Lys373/382 acetylation. To this end, we depleted HIPK2 by RNAi and subsequently treated the cells with a lethal dose of Adriamycin. HIPK2-depletion resulted in a massive reduction in DNA damage-induced p53 Lys382 acetylation (Figure 5a), in line with a previous report.^52^ As expected, also a substantial decrease in the HIPK2 phospho-targets SIRT1 Ser682 and in p53 Ser46^37, 39, 52^ was observed, and caspase-dependent PARP cleavage was absent after HIPK2 depletion (Figure 5a). These results highlight an essential role of HIPK2 in p53 acetylation and commitment to cell death.

We next sought to investigate whether HIPK2 regulates p53 acetylation through modulating SIRT1 function. As expected, co-expression of p53 and its acetyltransferase CBP resulted in p53 acetylation (Figure 5b). Ectopic expression of HIPK2 together with p53 and CBP potentiated p53 acetylation and also stimulated p53 Ser46 phosphorylation (Figure 5b), in accordance with a previous report.^39^ Furthermore, co-expression of wild-type SIRT1 in the presence of p53 and CBP led to an efficient reduction in p53 acetylation (Figure 5b), as reported previously.^7, 13^ Remarkably, ectopic expression of HIPK2 rescued p53 Lys382 acetylation in the presence of ectopically expressed SIRT1, indicating that HIPK2 inhibits p53 deacetylation by SIRT1 (Figure 5b). Of note, the rescuing effect of HIPK2 required the Ser682 residue of SIRT1, as HIPK2 failed to counteract SIRT1-dependent p53 deacetylation of a SIRT1^S682A^ point mutant (Supplementary Figure S4a). Thus, SIRT1 Ser682 phosphorylation is critical for p53 Lys382 acetylation by antagonizing SIRT1 function.

In line with our results, phosphorylation-mimetic SIRT1^S682D^ showed reduced p53 Lys382 deacetylation activity, whereas phosphorylation-deficient SIRT1^S682A^ deacetylated p53 to a comparable extent as wild-type SIRT1 protein (Figure 5c). This effect is specific for the SIRT1 Ser682 phosphorylation-site, as phosphorylation-mimetic SIRT1^S27D^ did not show this effect but behaved similar to wild-type SIRT1 (Supplementary Figure S4b). Taken together, Ser682 phosphorylation reduces SIRT1 deacetylase function and thus facilitates p53 acetylation.

We next reasoned that efficient p53 Lys382 acetylation upon DNA damage might require inhibition of SIRT1 function through phosphorylation at Ser682. Indeed, in the presence of phosphorylation-deficient SIRT1^S682A^ the DNA damage-mediated p53 acetylation by Adriamycin was decreased when compared with p53 acetylation levels in the presence of wild-type SIRT1 (Figure 5d). These findings indicate that Ser682 phosphorylation is important for efficient p53 acetylation upon DNA damage.

### SIRT1 Ser682 phosphorylation modulates p53-regulated gene expression and apoptosis

To test whether phosphorylation of SIRT1 at Ser682 regulates p53-dependent transcription, we performed Luciferase-reporter assays using the PUMA promoter. Whereas wild-type SIRT1 and phosphorylation-deficient SIRT1^S682A^ reduced p53-regulated transactivation of the PUMA promoter to a similar extend, phospho-mimetic SIRT1^S682D^ was less efficient in suppressing p53-dependent transcription (Figure 5e). In addition, RT-qPCR analysis of endogenous p53 target genes including p21, Bax, PUMA, Noxa and p53AIP1 revealed similar results (Figure 5e). Furthermore, phospho-mimetic SIRT1^S682D^ showed a slight stimulatory effect on the expression of the apoptotic p53 target genes PUMA, NOXA and p53AIP1 (Figure 5f).

Next, we explored the role of Ser682 phosphorylation in DNA damage-induced apoptosis. To this end, we transfected U2OS cells with empty vector, SIRT1 wild-type or SIRT1^S682D^ expression constructs and analysed DNA damage-induced apoptosis after Adriamycin-treated by FACS analysis. The level of Annexin V-positive cells in Adriamycin-treated cells transfected with empty vector was arbitrarily set to 100%. In contrast to wild-type SIRT1, which suppressed induction of apoptosis upon Adriamycin treatment, phospho-mimetic SIRT1^S682D^ instead enhanced apoptosis induction in response to Adriamycin treatment (Figure 5g). Taken together, phosphorylation of SIRT1 at Ser682 potentiates apoptotic p53 target gene expression and cell death.

### SIRT1 Ser682 phosphorylation regulates SIRT1–AROS interaction

Having shown that SIRT1 Ser682 phosphorylation inhibits SIRT1 deacetylase activity on p53, we aimed to identify the underlying molecular mechanism. To this end, we first analysed whether Ser682 phosphorylation directly affects the deacetlyase function of SIRT1 by performing *in vitro* deacetylation assays using bacterially expressed GST-SIRT1, GST-SIRT1^S682A^ and GST-SIRT1^S682D^. No differences in the deacetylase activity of these SIRT1 proteins on acetylated p53 and acetylated Histones were detectable *in vitro* (Supplementary Figure S5a, b), indicating that Ser682 phosphorylation does not directly affect SIRT1 deacetylase activity. Furthermore, no change in the subcellular localization was detectable (Supplementary Figure S5c), excluding the possibility that differential localization may account for decreased SIRT1 function.

**Figure 6:**
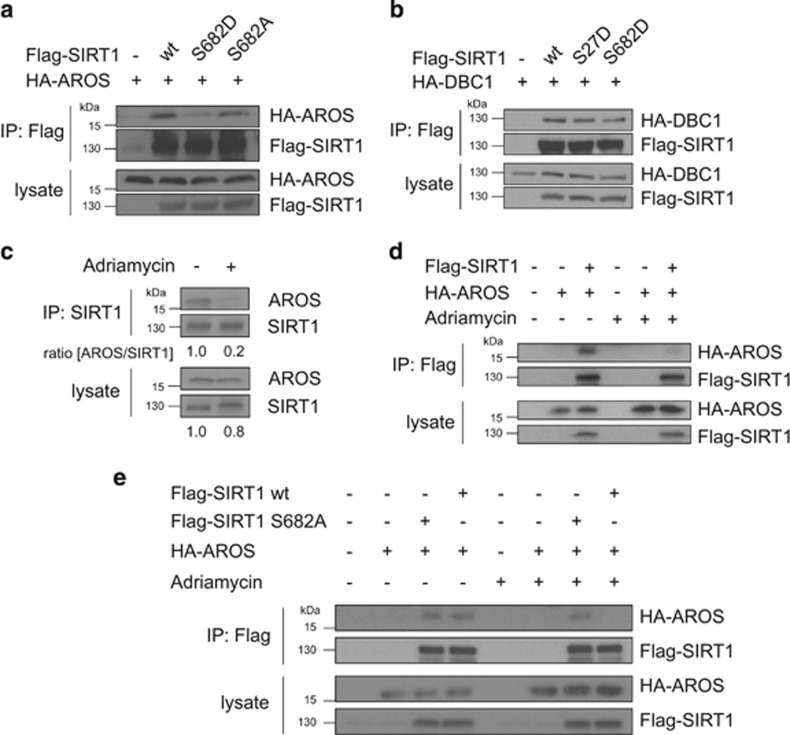
Regulation of the SIRT1–AROS complex by SIRT1 Ser682 phosphorylation and DNA damage. (**a**) SIRT1 Ser682 phoshorylation regulated SIRT1–AROS binding. 293T cells were transfected with the indicated expression constructs. Twenty-four hours after transfection Flag-SIRT1 was precipitated from the lysates and co-immunoprecipitation of AROS was analysed by immunoblotting using the indicated antibodies. Ten percent of the total cell lysate are shown as input control. (**b**) No effect of SIRT1 Ser682 phosphorylation on SIRT1–DBC1 interaction. 293T cells were transfected with the indicated expression constructs. Flag-SIRT1 was precipitated from the lysates and co-immunoprecipitation of AROS was analysed by immunoblotting using the indicated antibodies. Ten percent of the total cell lysate are shown as input control. (**c**) Disruption of the endogenous SIRT1–AROS complex after DNA damage. U2OS cells were treated with Adriamycin (0.75 *μ*g/ml for 4 h) and MG-132 (20 *μ*M) or left untreated. Subsequently, endogenous SIRT1 was precipitated from the lysates and co-immunoprecipitation of endogenous AROS was detected by immunoblotting. As control 10% of the total cell lysates were analysed. (**d**) Reduced interaction of ectopically expressed SIRT1 and AROS after DNA damage. HA-AROS and Flag-SIRT1 were expressed in 293T cells. Twenty-four hours after transfection cells were treated with Adriamycin (0.75 *μ*g/ml for 4 h) and MG-132 (20 *μ*M) or left untreated. Flag-SIRT1 was precipitated from the lysates and co-immunoprecipitation of HA-AROS was examined by immunoblotting. Ten percent of the total cell lysates are shown as input control. (**e**) Ser682 is required for dissociation of the SIRT1–AROS complex after DNA damage. HA-AROS and the phosphorylation-deficient mutant Flag-SIRT1^S682A^ were expressed in 293T cells. Cells were treated with Adriamycin (0.75 *μ*g/ml for 4 h) and MG-132 (20 *μ*M) or left untreated and Flag-SIRT1^S682A^ was precipitated from the lysates and analysed by immunoblotting using the indicated antibodies. Controls correspond to 10% of the total cell lysates

As SIRT1 activity is regulated by interaction with the activator AROS and the inhibitor DBC1,^19, 20, 21^ we analysed whether SIRT1–DBC1 or SIRT1–AROS interaction is modulated through phosphorylation of SIRT1 at Ser682. Strikingly, phospho-mimetic SIRT1^S682D^ showed a substantially reduced interaction with AROS when compared with wild-type and phospho-deficient SIRT1^S682A^ (Figure 6a). In contrast, interaction of SIRT1 with its inhibitor DBC1 was neither affected by SIRT1 Ser682 nor by SIRT1 Ser27 phospho-mimetic mutations (Figure 6b). These results suggest that SIRT1 phosphorylation at Ser682 modulates SIRT1–AROS binding.

### DNA damage triggers dissociation of the SIRT1–AROS complex

Next we investigated the regulation of the endogenous SIRT1–AROS complex. Whereas interaction of endogenous AROS and SIRT1 was clearly detectable in untreated cells, SIRT1–AROS interaction was substantially reduced 2 hours after Adriamycin treatment (Figure 6c). Comparable reduction in SIRT1–AROS binding after Adriamycin treatment was observed between ectopically expressed SIRT1 and AROS proteins (Figure 6d).

We next addressed whether Ser682 phosphorylation of SIRT1 is involved in dissociation of the SIRT1–AROS complex after DNA damage. Remarkably, the complex of AROS and phospho-deficient SIRT1^S682A^ remained stable upon Adriamycin treatment (Figure 6e). Our data indicate that (I) the SIRT1–AROS complex dissociates upon Adriamycin-induced DNA damage, and (II) that dissociation of the SIRT1–AROS complex is regulated by phosphorylation of SIRT1 at Ser682.

## Discussion

The SIRT1 deacetylase has an important role for cell fate regulation in response to DNA damage. Inhibition of SIRT1 function profoundly increases treatment responsiveness of cancer cells and cancer stem cells through facilitating an efficient p53 response.^7, 53^ Interestingly, SIRT1 inhibition can also trigger induction of apoptosis in the absence of exogenous DNA damage and of p53 through a Foxo4-dependent mechanism.^54^ Although these reports indicate that the inhibition of SIRT1 activity appears to be pivotal to elicit a productive cell death response, the cellular mechanisms by which severely damaged cells overcome the anti-apoptotic, p53-suppressive function of SIRT1 are largely unclear.

The apoptotic function of p53 is balanced by multiple mechanisms, and site-specific phosphorylation and acetylation are indispensable to unleash p53's death functions.^15, 17^ Here, we provide evidence that HIPK2, besides directly activating p53 through phosphorylation at Ser46 upon severe DNA damage,^38, 39^ also limits SIRT1 activity through direct phosphorylation to facilitate efficient p53 acetylation and cell death activation.

A previous study also described a role of HIPK2 in potentiating p53 Lys382 acetylation and linked this to SIRT1.^52^ However, it remained unclear how HIPK2 controls SIRT1 function. In addition, SIRT1–HIPK2 interaction was shown using ectopically expressed proteins.^55^ In this study the SIRT1 binding domain of HIPK2 has been assigned to the central part of HIPK2. Our results obtained from two independent mapping approaches (GST pulldown assays and coimmunoprecipitation analysis) support the conclusion that the interaction of HIPK2 with SIRT1 is mediated by its N-terminus and the kinase domain. The discrepancy between the results might be explained by the use of different HIPK2 deletion mutants, which presumably differ in protein folding/conformation and thus in their interaction profiles. In line with such an explanation, we have recently demonstrated that HIPK2 autointeracts and undergoes conformational changes.^36^

In the present study we demonstrate that HIPK2 regulates SIRT1 activity through direct phosphorylation of SIRT1 at Serine 682. Similar to HIPK2-mediated phosphorylation of p53 at Ser46, SIRT1 Ser682 phosphorylation is selectively triggered in the wake of severe DNA damage, which is tightly linked to the activation of pro-apoptotic p53 target gene expression and induction of cell death.

Our investigations on the molecular mechanism by which SIRT1 Ser682 phosphorylation regulates SIRT1 activity suggest a novel principle of SIRT1 regulation, which is control of SIRT1–AROS interaction. Ser682 phosphorylation of SIRT1 negatively regulates the interaction of SIRT1 with its activator AROS. In consequence, DNA damage, which triggers SIRT1 Ser682 phosphorylation, results in the dissociation of the SIRT1–AROS complex. Loss of SIRT1–AROS interaction is expected to result in a drop in SIRT1 activity, thereby facilitating efficient p53 acetylation upon DNA damage.

A recent study identified a role of AROS in ribosome biogenesis and ribosome function.^56^ AROS was shown to localize to nucleoli and nucleoplasmic ribosomes, and depletion of AROS resulted in reduction of 40 S ribosomal subunits and translating polysomes. It will be interesting to see whether AROS function is also affected in response to DNA damage.

**Figure 7:**
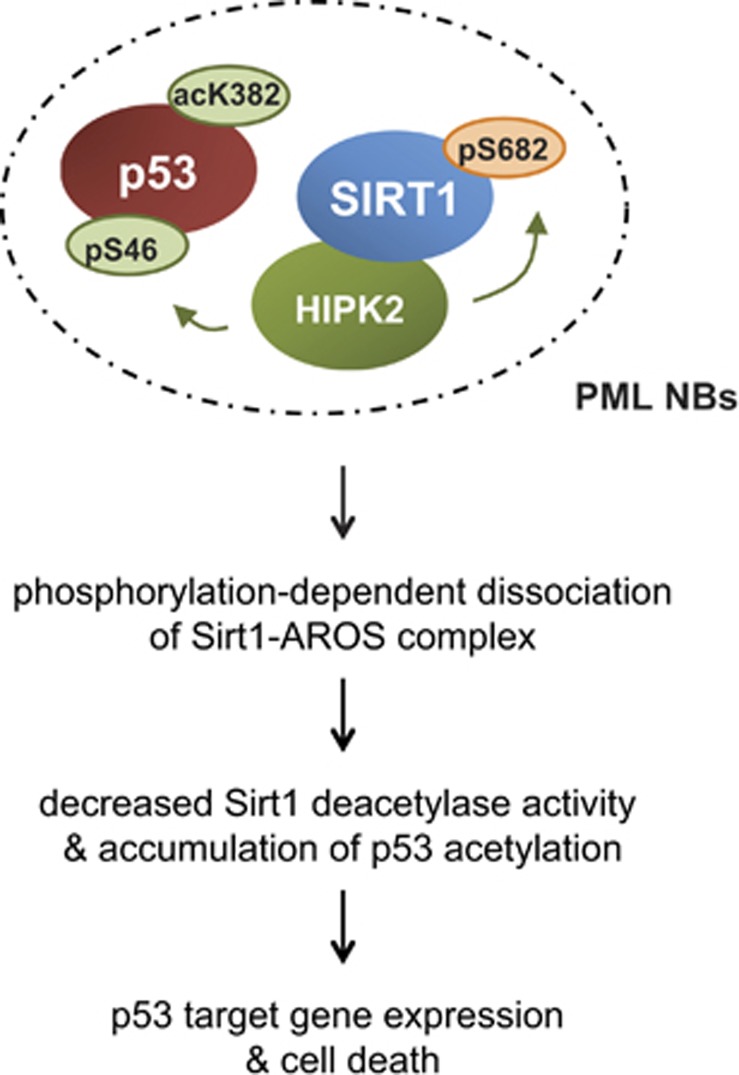
Proposed model for the interplay between HIPK2 and SIRT1 at PML-NBs upon severe genotoxic stress. Our data propose that in response to severe DNA damage SIRT1 and HIPK2 are recruited to PML-NBs by the PML isoform IV. Co-recruitment of both p53 regulatory enzymes facilitates crosstalk between SIRT1 and HIPK2 at the PML-NB. HIPK2 phosphorylates SIRT1, which in turn inhibits SIRT1 activity through dissociation of AROS. Reduced SIRT1 activity enables efficient p53 acetylation, expression of pro-apoptotic p53 target genes and potentiation of the DNA damage-induced cell death response

On the basis of our findings we propose the model that crosstalk between HIPK2 and SIRT1 at PML-NBs regulates SIRT1 activity in response to DNA damage (Figure 7). Upon damage, SIRT1 and HIPK2 form a complex in the cell nucleus at PML-NBs. Using the PML-NB as a meeting place, HIPK2 phosphorylates SIRT1 at Ser682 in response to severe DNA damage. This principle of SIRT1 regulation might facilitate fine tuning of SIRT1 activity through integrating diverse cellular signals at PML-NBs. Along these lines, PML-NBs have been shown to sense and to react to numerous kinds of cellular stress signals including cytokines, DNA damage, oncogenic fusion proteins, heat shock, heavy metals and viral infection.^43, 46, 57^ As numerous key enzymes of the genotoxic stress response including checkpoint kinases ATM,^58^ ATR^59^ and Chk2^60^ and the deubiquitinase USP7/HAUSP^61^ are recruited to PML-NBs upon stress, we speculate that additional crosstalk might occur at PML-NBs. Furthermore, as cellular stress has an impact on the number, size and composition of PML-NBs,^45^ it is tempting to speculate that crosstalk at PML-NBs balances the ‘output signals' such as p53 posttranslational modifications, which finally determine the cellular response.

Previous studies demonstrated that genetic and pharmacological inhibition of SIRT1 results in increased induction of p53-dependent apoptosis, both in somatic cancer cells and in cancer stem cells.^3, 62^ These findings make SIRT1 a promising target in cancer therapy. However, studies performed in cancer cells and in mice indicate that SIRT1 is also essential for maintenance of genomic stability, tumour suppression and DNA repair.^4, 58^ Thus, it could be challenging in the future to define conditions to pharmacologically modulate SIRT1 function in such a manner that it will be beneficial for cancer therapy. In light of these findings, it will be important to categorize tumours based on their p53 status before treatment with SIRT1 inhibitors and to design approaches that allow specific intervention with the anti-apoptotic activities of SIRT1. In this context it might be promising to exploit cellular mechanisms facilitating SIRT1 inactivation, such as the HIPK2-SIRT1-AROS pathway reported here.

## Materials and Methods

### Cell lines, cell culture, transduction and transfection

U2OS, 293 T, H1299 (obtained from ATCC, Manassas, VA, USA) were cultured in DMEM, 4.5 g/ml glucose (Gibco, Life Technologies GmbH, Darmstadt, Germany) supplemented with 10% FCS, 1% (w/v) penicillin/streptomycin, 1 mM sodium pyruvate and 20 mM Hepes buffer at 37 °C and 5% CO_2_. Cells were transfected with either CaPO_4_ precipitation method or Lipofectamine 2000 (Life Technologies, Darmstadt, Germany) and the indicated expression constructs. U2OS cells were transduced with a retroviral control plasmid pGLentiLox3.7-shLuci or pGLentiLox3.7-shSIRT1 to knock down SIRT1 by retroviral transduction after having produced the virus in 293T cells.

### Antibodies

The following antibodies were used: mouse monoclonal SIRT1 7B7 (Novus Biologicals, LLC, Littleton, CO, USA) for immunofluorescence staining and mouse monoclonal SIRT1 Clone 3H10.2 (Millipore, Merck KGaA, Darmstadt, Germany), rabbit polyclonal SIRT1 (M07-131; Millipore, Merck KGaA) for immunoblotting, RPS19BP1 (AROS, ATLAS Antibodies AB, Stockholm, Sweden), PARP and p53 phospho-Ser46 (BD Pharming, Pharmingen, San Diego, CA, USA), p53 DO-1, GFP FL and PML H-238 (Santa Cruz Biotechnologies Inc., Dallas, TX, USA), Flag M2 (Sigma-Aldrich, St Louis, MO, USA), actin C4 (MP Biomedicals, (LLC, Santa Ana, CA, USA)), HA clones 12CA5 and 3F10 (Roche, F. Hoffmann-La Roche Ltd., Basel, Switzerland), p53 phospho-Ser46, SIRT1 (rabbit Ab), SIRT1 phospho-Ser27 (all Cell Signaling Technologies Inc., Danvers, MA, USA), acetyl-Lys373/382 p53 (Millipore, Merck KGaA). Affinity-purified rabbit HIPK2 antibodies have been described previously.^33, 39^ Phospho-specific SIRT1 pSer682 antibodies were generated by immunizing rabbits with the following KLH-coupled peptide: NH_2_-SGTCQ(pS)PSLEC-CONH_2_. Rabbit sera were affinity-purified against the phospho-peptide and subsequently non-phosphorylation specific antibodies were removed by a column containing the immobilized non-phospho-peptide. The rabbit SIRT1 phospho-Ser682 antibody will be commercially available soon from Millipore (Merck KGaA).

### Expression constructs

Human HIPK2 constructs have been described previously.^33, 63^ SIRT1 expression construct were kindly provided by Robert Weinberg and Renate Voit or generated by standard PCR technology. SIRT1 point mutant constructs were generated by standard PCR. p53 constructs were kindly provided by Wei Gu and Giannino del Sal. DBC1 cDNA was obtained from Invitrogen (Life Technologies GmbH) as Gateway system vector and cloned into destination vectors. AROS cDNA was chemically synthesized (GeneArt Gene Synthesis, Thermo Fisher Scientific Inc., Waltham, MA, USA) and expression constructs were generated by standard DNA cloning. All constructs used in this study were verified by DNA sequencing.

### GST pulldown assays

GST fusion protein expression, protein purification and GST-pulldown assays were performed essentially as described.^33^ In brief, GST fusion proteins were expressed in the *E.coli* BL21 strain and purified using glutathione (GSH) sepharose 4B beads (GE Healthcare, GE Healthcare UK Limited, Buckinghamshire, England). 0.2–0.4 *μ*g of bead-coupled GST fusion proteins were used for the pulldowns. GST fusion proteins were incubated with proteins generated by *in vitro* translation using the TNT Coupled Reticulocyte Lysate System (Promega Corporation, Madison, WI, USA). GST-SIRT1, ^35^S-Flag-HIPK2 pull down was performed in 20 mM Tris-HCl, pH7.4, 0.05% NP40, whereas GST-HIPK2, ^35^S-Flag-SIRT1 pull down in 1 × PBS, 0.05% NP40.

### Immunoblotting and immunoprecipitation

Immunoprecipitation and immunoblotting were performed as described previously and proteins were detected by Western Lightning Plus-ECL (PerkinElmer, Waltham, MA, USA) and enhanced chemiluminescence SuperSignal West Dura and Femto (Pierce, Life Technologies GmbH).^33^ SIRT1-HIPK2 coimmunoprecipitation was performed in lysis buffer (20 mM Tris–HCl pH 7.4, 10% glycerol, 1% NP40, 150 mM NaCl, 25 mM NaF, 5 mM EDTA, 0.1% SDS, 1 mM sodium vanadate, 1 × complete protease inhibitor (Roche, F. Hoffmann-La Roche Ltd.), 100 *μ*M MG-132). SIRT1–AROS and SIRT1–DBC1 co-immunoprecipitation were performed as described elsewhere.^20, 21^ Immunoprecipitations were performed using the Rabbit IgG and the Mouse IgG TrueBlot System (eBioscience Inc., San Diego, CA, USA) or Anti-Flag M2-Affinity Gel (Sigma-Aldrich) or GFP-trap (ChromoTek GmbH, Planegg-Martinsried, German) according to the manufacturer's instructions. Immunoprecipiatation reactions were incubated for 2 h at 4 °C on a rotating wheel and washed three times in lysis buffer. Samples were denatured at 95 °C for 5 min, separated by SDS–PAGE on 8%, 9.5%, 13% SDS gels or on 4–15% Mini-PROTEAN TGX Precast Gels (BioRad, Hercules, CA, USA) and analysed with the indicated antibodies.

### RT-qPCR analysis

Total RNA was collected from cultured cells using TRIzol reagent (Invitrogen, Life Technologies GmbH). After RNA extraction, cDNA was synthesized using High capacity RNA to cDNA kit (Applied Biosystems, Life Technologies GmbH). Primers were used with TaqMan Gene Expression to perform quantitative (real-time) qRT-PCR (Applied Biosystems, Life Technologies GmbH) using a Step-One-Plus real-time PCR system (Applied Biosystems, Life Technologies GmbH). PCR conditions were as follows: step 1: 95 °C for 10 min; step 2: 40 cycles of 95 °C for 15 s followed by 60 °C for 1 min. The following primers were used: *CDKN1A (Hs00355782)*, *BBC3 (Hs00248075)*, *BAX (Hs00180269)*, *PMAIP1 (Hs00560402)*, *TP53AIP1 (Hs00223141)*, *HPRT1 (Hs02800695)* (Applied Biosystems, Life Technologies GmbH). HPRT1 expression was used as an endogenous control gene to normalize input cDNA. Ratios of the expression level of each gene to that of the reference gene were calculated using the delta-delta-CT-method.

### RNA interference

For HIPK2 knockdown either stealth siRNA against human HIPK2 (LifeTechnologies) was used or a conventional HIPK2 siRNA (QIAGEN Sciences, Germantown, MD, USA). For PML depletion the siPML SmartPool (Dharmacon) was used. For control experiments the medium GC-content control stealth siRNA (LifeTechnologies) and a GL2 luciferase siRNA (QIAGEN Sciences) was used. siRNA duplexes (final concentration 100 nM) were transfected using Lipofectamin 2000 (LifeTechnologies) as specified by the manufacturer.

### Immunofluorescence microscopy

Cells were seeded onto coverslips and treated or transfected with the indicated constructs. Cells were fixed with 4% PFA in PBS for 40 min at RT. After washing once with PBS, the cells were blocked in 10% goat serum in PBS for 1 h at RT. Cells were incubated with the following antibodies for 1 h at 20 °C as indicated in the figure legend: rabbit anti phospho-Ser682 SIRT1, rabbit anti-HIPK2, mouse anti-SIRT1, Flag (M2, Sigma-Aldrich). After washing with PBS, cells were incubated with secondary antibodies AlexaFluor488 or AlexaFlour594 donkey anti-rabbit (Invitrogen, Life Technologies GmbH) in PBS with Hoechst 1:1000 w/v (Sigma-Aldrich) and mounted on glass slides with Mowiol (Sigma-Aldrich). Images were taken using a confocal laser scanning microscope (FluoView1000, Olympus, Hamburg, Germany) with a × 60 oil objective using the sequential scanning mode. All images were collected and processed using the FluoView Software (Olympus) and ImageJ (National Institutes of Health, Bethesda, MD, USA; http://imagej.nih.gov/ij/).

### DNA damage

Cells were incubated with culture medium supplemented with adriamycin (stock dissolved in water) at the specified concentrations for indicated time points. After incubation cells were harvested and processed as indicated.

### Apoptosis measurement

Cell were transfected with indicated expression constructs and apoptosis was determined by FACS analysis (Becton, Dickinson and Company, Franklin Lakes, NJ, USA) of 100 000 cells using Annexin V-FITC Apoptosis Detection Kit (Sigma-Aldrich) according to the manufacturer's instructions.

### *In vitro* SIRT1 deacetlyase activity assay

*In vitro* deacetylase assays were carried out by using human recombinant GST-SIRT1 wild-type and mutant proteins that were expressed in *E.coli* BL21 and purified by standard procedures under non-denaturing conditions as previously described.^33^ Subsequently, proteins were eluted by standard protocols. To compare the activity of GST-SIRT1 (0.5–1 ng) and mutant GST-SIRT1^S682D^ (0.5–1 ng) the SIRT1 Direct Fluorescent Screening Assay Kit (Cayman Chemical Company, Ann Arbor, MI, USA) was used according to the manufacturer's instruction. 0.5–1 ng GST–SIRT1 and 0.5 ng Histone preparations^53^ were used for deacetylase reactions. Reactions were incubated at 30 °C for 10–30 min. Proteins were separated on 13 or 8% SDS–PAGE, transferred to PVDF membrane and detected by immunoblotting.

### *In vitro* kinase assays

*In vitro* kinase assays were performed in principle as described previously.^64^ 0.2–0.5 *μ*g bacterially expressed and purified 6 × His-HIPK2 proteins or GST-HIPK2 (amino acids 1–551) were incubated in 30 *μ*l kinase buffer containing 40 *μ*M cold ATP and 5 *μ*Ci [γ-^32^P] ATP and 1-2 *μ*g GST-SIRT1 protein. After incubation for 30 min at 30 °C, the reaction was stopped by adding 5 × SDS loading buffer. After separation by SDS–PAGE, gels were fixed, dried and exposed to X-ray films.

### Luciferase reporter assay

H1299 cells were transfected with empty vector (pCMV3Tag1A) or the indicated expression plasmids. Twenty-four hours after transfection cells were harvested and the Luciferase Assay was performed using the Dual-Luciferase Assay System (Promega Corporation) according to the manufacturers' instructions.

## Abbreviations

AROS: active regulator of SIRT1
DBC1: deleted in breast cancer 1
GFP: green fluorescent protein
GST: glutathione*-S*-transferase
HIPK2: homeodomain interacting protein kinase 2
PML: promyelocytic leukaemia protein
PML-NB: PML nuclear body
SIRT1: sirtuin 1
