# Supplementary figures and images for: HIPK2 restricts SIRT1 activity upon severe DNA damage by a phosphorylation-controlled mechanism
Source: Cell Death Differ. 2015 Jun 26;23(1):110–22. doi: 10.1038/cdd.2015.75 (PMC4815982; doi:10.1038/cdd.2015.75)

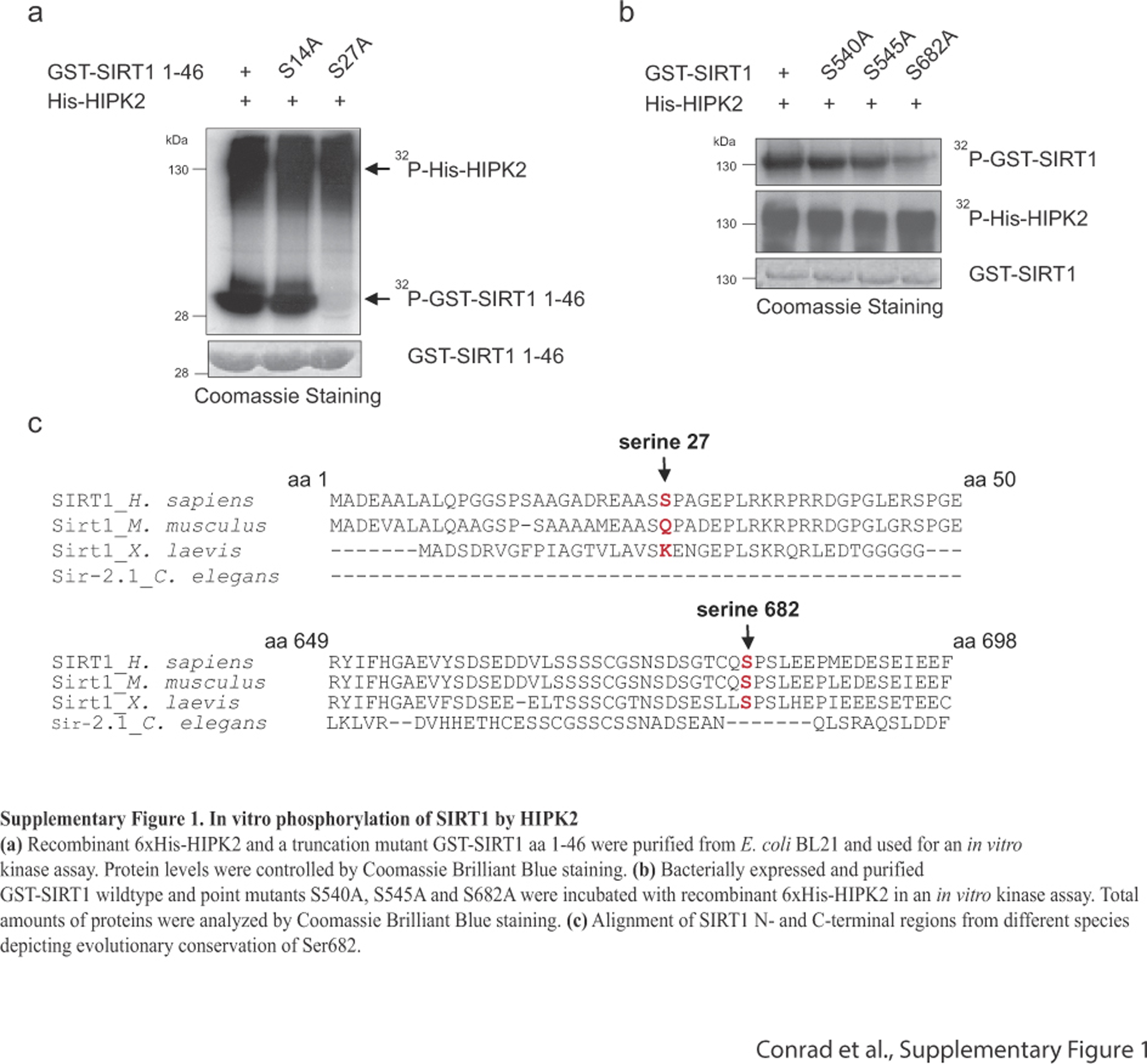

Supplement: Supplementary Figure S1 [file cdd201575x1.tif]

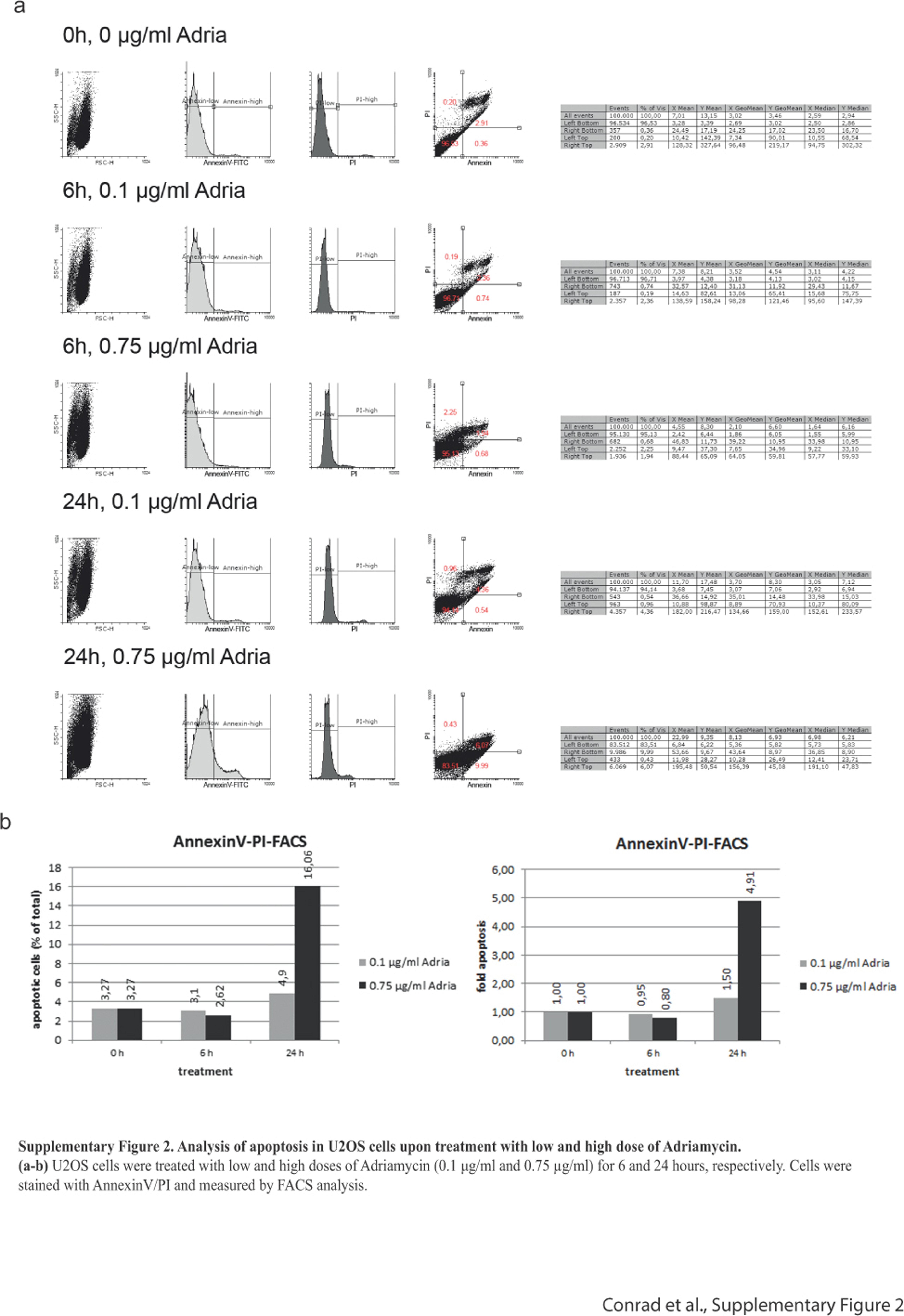

Supplement: Supplementary Figure S2 [file cdd201575x2.tif]

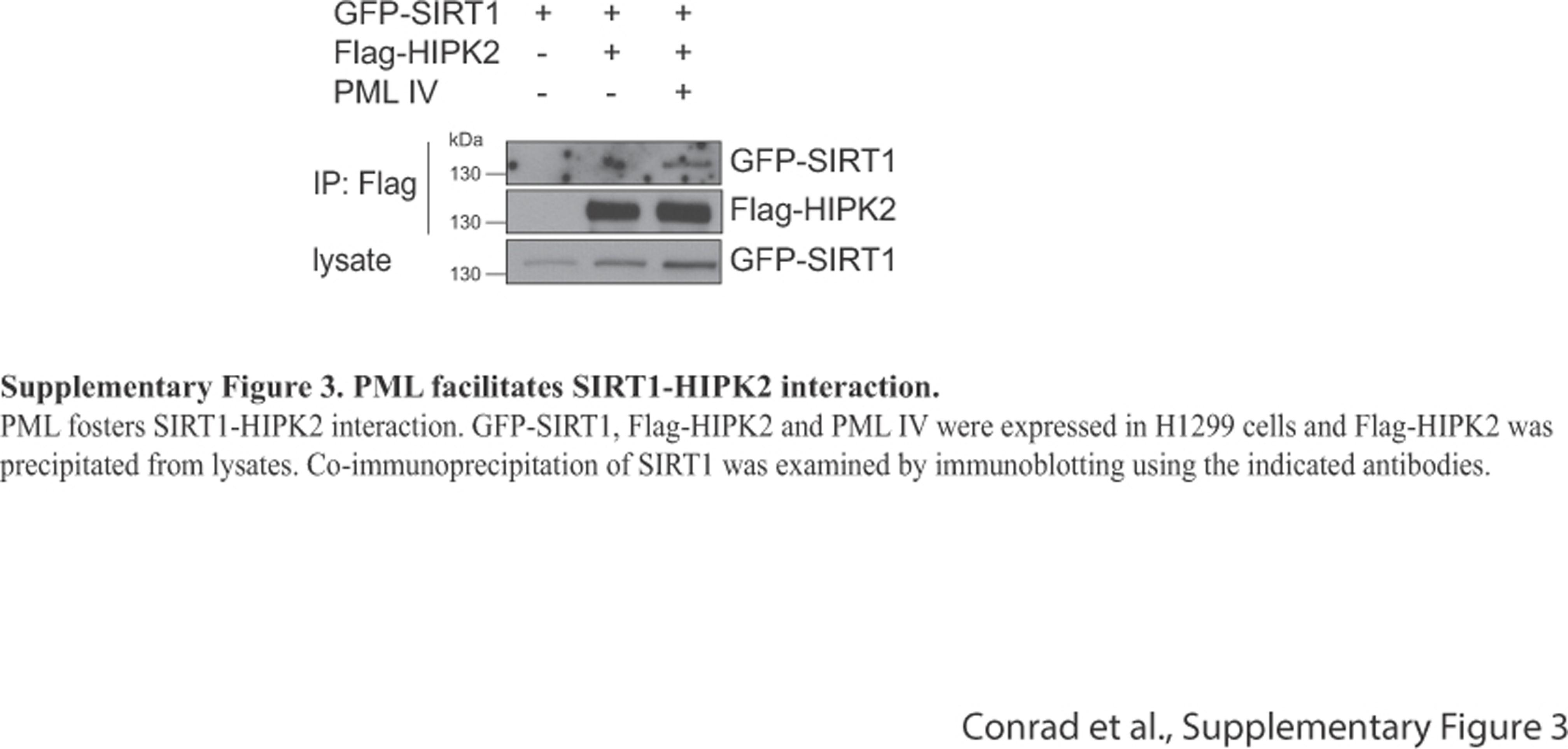

Supplement: Supplementary Figure S3 [file cdd201575x3.tif]

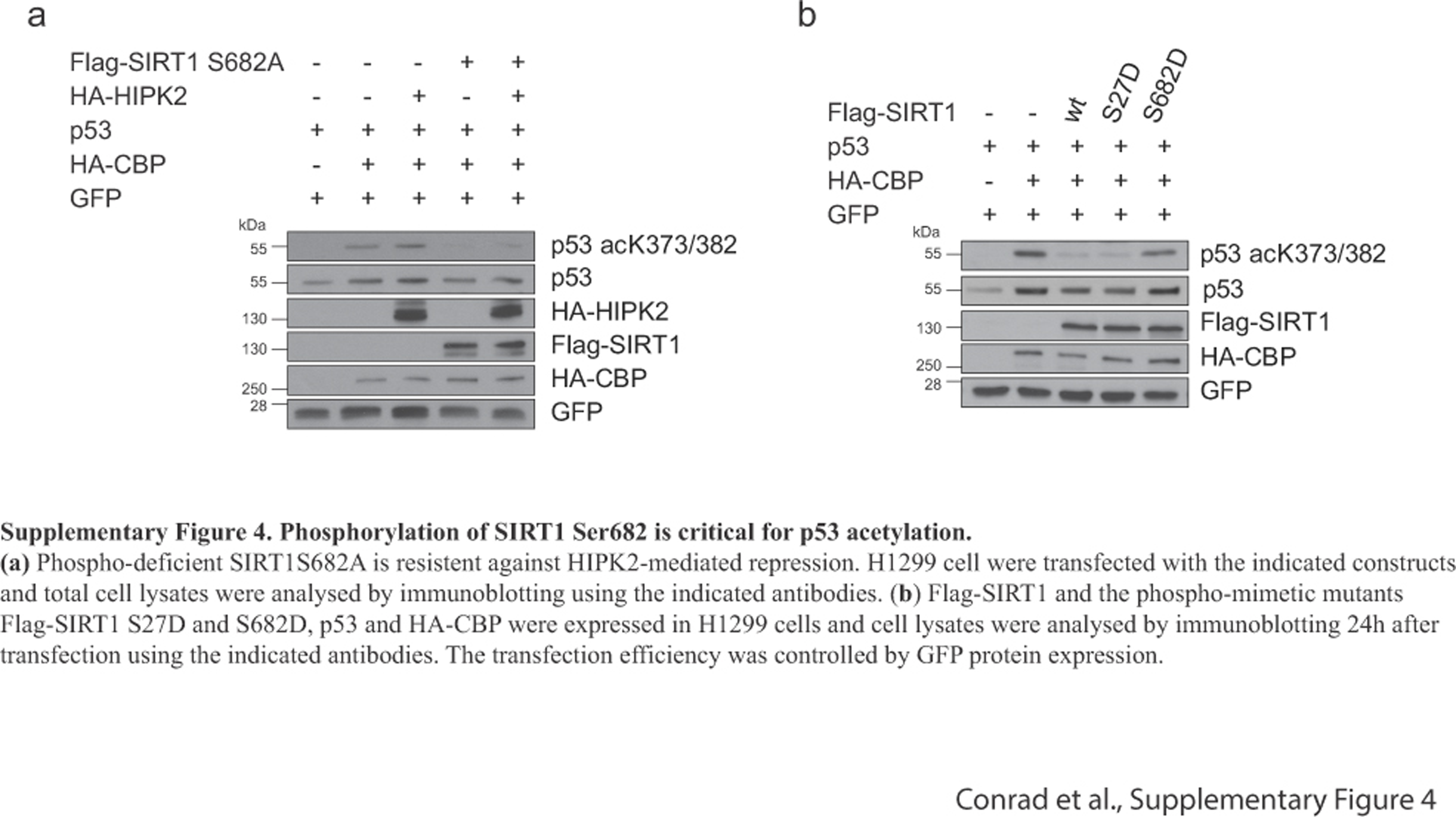

Supplement: Supplementary Figure S4 [file cdd201575x4.tif]

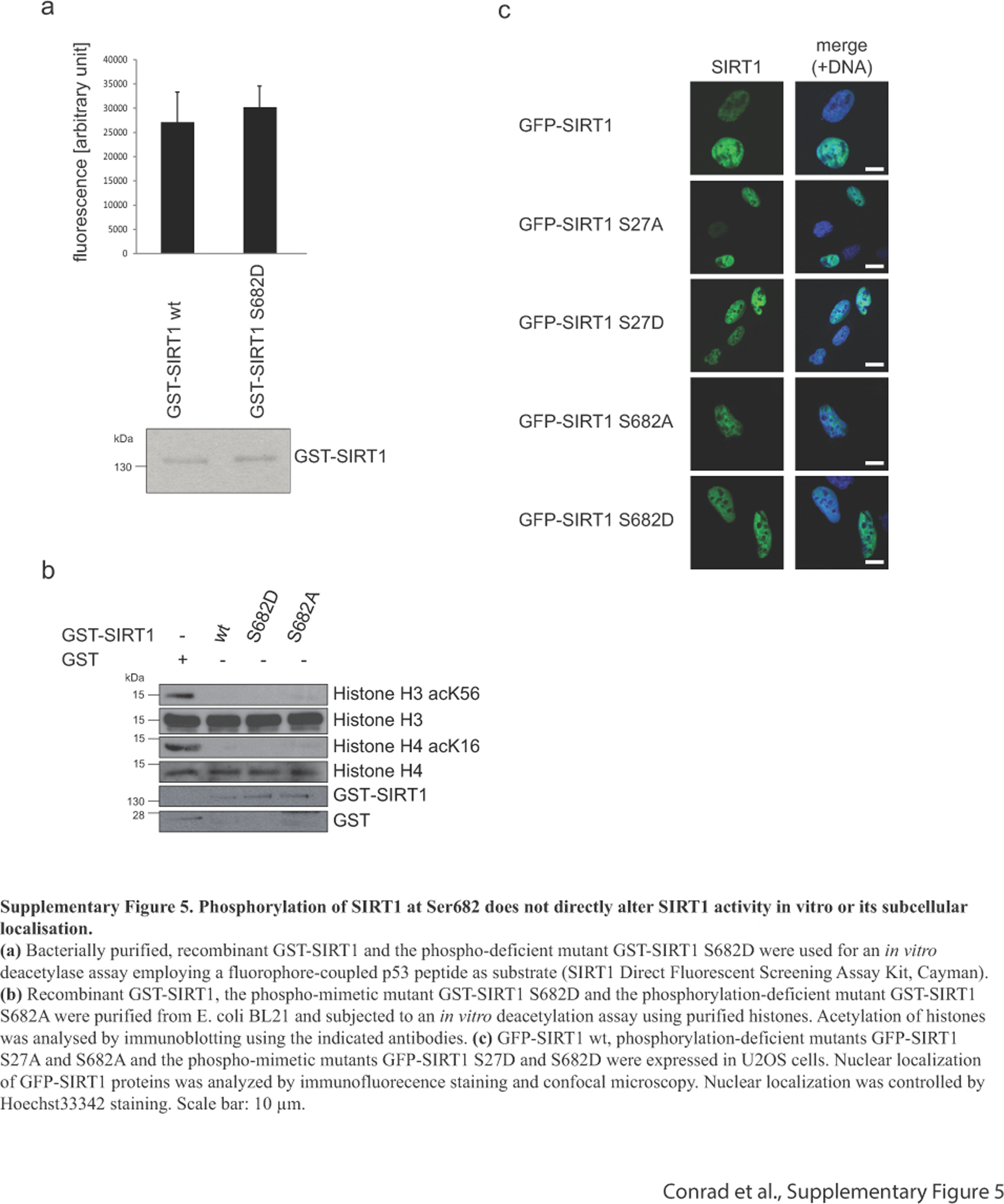

Supplement: Supplementary Figure S5 [file cdd201575x5.tif]
